# Multimodal Non-Contact Luminescence Thermometry with Cr-Doped Oxides

**DOI:** 10.3390/s20185259

**Published:** 2020-09-15

**Authors:** Vitaliy Mykhaylyk, Hans Kraus, Yaroslav Zhydachevskyy, Volodymyr Tsiumra, Andriy Luchechko, Armin Wagner, Andrzej Suchocki

**Affiliations:** 1 Diamond Light Source, Harwell Campus, Didcot OX11 0DE, UK; armin.wagner@diamond.ac.uk; 2 Denys Wilkinson Building, Department of Physics, University of Oxford, Oxford OX1 3RH, UK; hans.kraus@physics.ox.ac.uk; 3 Institute of Physics, Polish Academy of Sciences, Al. Lotników 32/46, 02-668 Warsaw, Poland; zhydach@ifpan.edu.pl (Y.Z.); tsiumra@gmail.com (V.T.); suchy@ifpan.edu.pl (A.S.); 4 Lviv Polytechnic National University, 12 Bandera, Lviv 79646, Ukraine; 5 Ivan Franko National University of Lviv, Tarnavskogo Str. 107, 79017 Lviv, Ukraine; andriy.luchechko@lnu.edu.ua; 6 Institute of Physics, University of Bydgoszcz, Weyssenhoffa 11, 85-072 Bydgoszcz, Poland

**Keywords:** non-contact luminescence thermometry, luminescence decay thermometry, intensity ratio thermometry, wavelength shift thermometry, Cr^3+^ emission

## Abstract

Luminescence methods for non-contact temperature monitoring have evolved through improvements of hardware and sensor materials. Future advances in this field rely on the development of multimodal sensing capabilities of temperature probes and extend the temperature range across which they operate. The family of Cr-doped oxides appears particularly promising and we review their luminescence characteristics in light of their application in non-contact measurements of temperature over the 5–300 K range. Multimodal sensing utilizes the intensity ratio of emission lines, their wavelength shift, and the scintillation decay time constant. We carried out systematic studies of the temperature-induced changes in the luminescence of the Cr^3+^-doped oxides Al_2_O_3_, Ga_2_O_3_, Y_3_Al_5_O_12_, and YAlO_3_. The mechanism responsible for the temperature-dependent luminescence characteristic is discussed in terms of relevant models. It is shown that the thermally-induced processes of particle exchange, governing the dynamics of Cr^3+^ ion excited state populations, require low activation energy. This then translates into tangible changes of a luminescence parameter with temperature. We compare different schemes of temperature sensing and demonstrate that Ga_2_O_3_-Cr is a promising material for non-contact measurements at cryogenic temperatures. A temperature resolution better than ±1 K can be achieved by monitoring the luminescence intensity ratio (40–140 K) and decay time constant (80–300 K range).

## 1. Introduction

Luminescence thermometry is a non-contact temperature measurement technique that utilizes emission characteristics that exhibit a temperature dependence [1]. Temperature can influence a number of different luminescence characteristics, such as shapes of emission spectra, intensity, or luminescence decay time constants. This makes a range of suitable luminescence parameters available for use in temperature monitoring [2,3]. When compared with other techniques, luminescence thermometry offers the capability of remote temperature sensing, which is a crucial advantage in comparison with conventional contact thermometers. Accurate temperature sensing in a way that does not interfere with the system is a key feature, which enables applications of micro thermometry for measurements of intracellular temperature fluctuations and temperature mapping of microcircuits or microfluids (see review [4,5] and references). Additionally, it is an essential requirement for measurements on objects in harsh environments (i.e., very hot or cold, aggressive chemicals, strong electromagnetic field) where direct contact with the object is limited due to it requiring isolation, shielding, or a lack of immobility. Therefore, luminescence thermometry has become an important part of metrology and is evolving rapidly. Evidence for that is the fact that more than 100 luminescence materials for this application have already been identified and reported (see reviews [6,7,8] and references therein) while new prospective compounds are emerging on a regular basis [9,10,11,12,13,14,15].

The materials typically used in luminescence thermometry (often referred to as thermographic phosphors) are rare-earth-doped, transition-metal-doped compounds [16,17,18] or transition metal oxides exhibiting intrinsic emission [19]. In these materials, the luminescence characteristics with sensitivity to temperature changes are emission peak energy, intensity, and lifetimes of excited states. The thermally-induced change of the emission peak is due to the interaction of electronic states with phonons. The effect is pronounced in materials doped with transition metals where the peak wavelength of luminescence due to transitions within d-shells exhibits a noticeable shift with temperature [20]. The intensity-based method normally identifies two transitions and compares the ratio of their emission intensities. The intensity of each transition is proportional to the population of an excited state, which obeys Boltzmann statistics and, therefore, is temperature-dependent. In this way, the temperature variation of the luminescence intensity ratio can be used to measure absolute temperature [21]. The advantage of this approach is that the luminescence intensity ratio is not affected by variation in the emission intensity in contrast to intensity-based methods. An alternative technique that offers additional benefit is based on luminescence lifetime measurements, which utilizes the temperature dependence of the luminescence decay time constant [22]. These measurements are virtually not affected by scattered light, background emission, or intensity fluctuations, which translates into better accuracy and enables the possibility of obtaining temperature mapping with high spatial resolution [23,24]. 

Altogether, luminescence non-contact methods offer reliable and accurate measurements of temperature with high spatial and temporal resolution over a wide temperature range from cryogenic temperatures up to almost 2000 K [6,18]. Nonetheless, non-contact luminescence thermometry is used primarily for measurements around or above ambient temperature. Application to measurements at cryogenic temperatures has been rather infrequent, even though it offers an important advantage over conventional techniques, i.e., the response of a luminescence sensor is not affected by heat leaks and thermal contacts, which are the main causes of poor accuracy at cryogenic temperatures. Thus, this provides a reliable estimate of the true temperature. A number of feasibility studies of luminescence lifetime thermometry demonstrated the suitability of La_2_O_2_S-Eu [25], Mg_4_FGeO_6_:Mn [24], Yb-doped CaF_2_, and SrF_2_ [26] as well as lanthanide compounds [27] for measurements in cryogenic environments.

Strong interest in this application of the technique emerged recently, driven by the necessity of having to monitor the temperature of biological samples in experiments that use strong ionizing radiation from synchrotron light sources to study properties of matter. To protect the samples from radiation damage, they need to be kept frozen below the temperature of the phase transition from vitreous to crystalline ice, typically occurring above 130 K [28]. When heated above this temperature, trapped radicals produced by X-ray absorption become mobile and damage the biological samples. Furthermore, there is increasing evidence that, with some experiments, the energy deposited by the synchrotron beam can cause a significant increase of temperature in samples of microscopic size [29,30,31,32]. As technology at synchrotron rings and beamlines evolves, radiation-induced heating of experimental samples is a concern, and research began monitoring and quantifying this effect.

Luminescence thermometry is well suited for this, and investigations in this direction have started a few years ago at the Diamond Light Source. A first dedicated luminescence thermometry system for non-contact temperature monitoring in a vacuum environment was developed and deployed at the I23 beamline [33,34]. The temperature is derived from changes in the luminescence decay characteristics of a Bi_4_Ge_3_O_12_ (BGO) scintillation sensor. Results from extensive testing demonstrated the reliability and accuracy as well as the advantages of the technique. The uncertainty of temperature determination over the 10–270 K temperature range was shown to be ±1.6 K. A complementary approach has been adopted at the I24 beamline where the effect of radiation-induced heating was investigated by monitoring the shift of the emission wavelengths in a ruby (Al_2_O_3_-Cr) crystal [35]. The maximum temperature increase of the ruby sensor during exposure to an X-ray beam was found to be 58 K. More recently, the promising concept of fast luminescence decay thermometry was tested using ultrafast scintillations of PbI_2_ [36]. It was shown that changes in the decay time of PbI_2_ can be measured by using the pulsed structure of synchrotron radiation and permitting temperature monitoring at a sampling rate of megahertz. All these examples demonstrate that the non-contact luminescence method of temperature monitoring is developing rapidly, which adds to the toolset commonly used with synchrotron instrumentation.

## 2. Background

There is a strong case for identifying additional materials with luminescence properties suitable for temperature sensing at cryogenic temperatures. Application in the low-temperature range focuses the search onto systems with thermally induced processes, where processes of reorganization of population of excited states and thermal quenching require a low activation energy. This can then translate into changes of a luminescence parameter with temperature, as observed for the luminescence lifetime in tungstates and molybdates [37], intensity ratio of emission lines in rare earth RE^3+^ doped Y_2_O_3_ [21], or emission line shift in Ga-doped ZnO [38]. Among many materials used for non-contact luminescence thermometry, Cr^3+^-doped compounds are particularly interesting since luminescence of Cr^3+^ exhibits a variety of temperature-dependent effects. The reason for this is due to the energy structure of the Cr^3+^ ion and its sensitivity to the crystal field. The ground state of Cr^3+^ is ^4^A_2_ while the lowest excited levels are split by the crystal field into ^4^T_2_ and ^2^E levels. In materials with a strong crystal field, the emission is dominated by the transition from the ^2^E state, which is split further into $\bar{E}$ and $\bar{2A}$ levels to the ground state. These transitions give rise to two sharp emission lines, R_1_ ($\bar{E}$→^4^A_2_) and R_2_ ($\bar{2A}$→^4^A_2_), observed in the deep red spectral range with the exact wavelength being material-dependent [39]. The emission characteristics of Cr^3+^ ions are strongly influenced by the strength of the crystal field and thermal quenching effects [40,41]. In particular, the effect of temperature manifests itself explicitly in energy shifts of the R-lines, changes to their intensities, and luminescence decay time [39]. 

Due to these clearly identifiable features of the luminescence that change with temperature, Cr^3+^-based materials received significant attention for thermometric application. Among them, Al_2_O_3_-Cr is the most comprehensively examined and widely used material in a number of optical sensor schemes. The prospect of this material for temperature sensing has been recognised by following studies of the temperature-induced changes of R-lines in Cr-doped Al_2_O_3_ [42]. This prompted the development of luminescence thermometry based on a wavelength shift [43,44] and intensity ratio [45,46] of R-lines. Research into ruby-based luminescence lifetime thermometry resulted in good precision and reliability [47] and, thus, the method is used for temperature monitoring over a relatively wide range [48,49,50,51,52]. The feasibility of other Cr-doped luminescence sensors for luminescence thermometry has been shown in many other materials, including garnets Y_3_Al_5_O_12_ [53], Gd_3_Al_5_O_12_ [54], perovskites YAlO_3_ [55], GdAlO_3_ [56], alexandrite BeAl_2_O_4_, [57], spinel MgAl_2_O_4_ [58], borates YAl_3_(BO_3_)_4_ [59], gallates Bi_2_Ga_4_O_9_ [60], aluminates LiAl_5_O_8_ [61], etc. 

Although the main emphasis of research and development so far was on materials for non-contact thermometry in the high-temperature domain, there are some earlier studies in which the feasibility of Al_2_O_3_-Cr luminescence sensors for measurements below 100 K has been investigated [45,49,62]. In these works, different temperature-sensing scenarios were explored. It has been found that the sensitivity of the methods based on an energy shift and luminescence decay time of R-lines in Al_2_O_3_-Cr reduces dramatically below 100 K since both parameters exhibit little or no change at further cooling. At low temperatures, the mechanisms responsible for changes of the energy position of the emitting level and its decay time, such as electron-phonon interaction and thermal depopulation of ^2^E emitting levels, respectively, are suppressed in the crystal. The intensity ratio of R-lines, however, exhibits a strong change with temperature below 100 K. The population of the emitting states described in thermal equilibrium by the Boltzmann statistics changes prominently at low temperatures due to the small energy splitting of the levels (a few meV). Evidently, because of high sensitivity, this is a very attractive method for monitoring at cryogenic temperatures. 

It has become clear that, when considering these fundamental properties of the Cr^3+^ emission together, the possibility for multimodal temperature sensing is clear. Different luminescence characteristics of materials can be monitored with each providing a measure of temperature in the temperature domain where its sensitivity is high. This approach should pave the way for the application of a Cr-doped luminescence material for non-contact temperature monitoring over a wider low-temperature range. An important advantage of the multimodal sensor is the possibility of cross-referencing, which improves the reliability and accuracy of the measurements [8]. In this work, by focusing on Cr-doped materials, we carried out a systematic investigation of luminescence characteristics in Al_2_O_3_-Cr, Ga_2_O_3_-Cr, YAlO_3_-Cr, and Y_3_Al_5_O_12_-Cr as a function of temperature over the 4–300 K range. Ruby is a well-known standard and, therefore, serves as a benchmark in temperature sensing while Ga_2_O_3_-Cr and YAlO_3_-Cr and Y_3_Al_5_O_12_-Cr were selected based on results from prior tests that indicated their potential for application as multimode sensors at cryogenic temperatures. 

## 3. Methods 

All samples of Al_2_O_3_-Cr, Ga_2_O_3_-Cr, YAlO_3_-Cr, and Y_3_Al_5_O_12_-Cr had a nominal concentration of Cr^3+^ ions of 0.05% with respect to the substituted (Al or Ga) ions. A low concentration was chosen to minimize the impact of energy transfer and reabsorption processes. This ensures that a single-exponential model for the decay can be used while avoiding the complexity and uncertainty that multi-exponential models bring with them. α-Al_2_O_3_-Cr and β-Ga_2_O_3_-Cr were in the form of bulk single crystals, grown by the Czochralski technique or the floating zone technique with radiation heating, respectively. For the spectroscopic measurements, these single-crystalline materials were prepared in the form of slices of about 100-μm thickness. Two other materials (YAlO_3_-Cr and Y_3_Al_5_O_12_-Cr) were prepared in the form of microcrystalline powders synthesized by the sol−gel technique and, subsequently, calcined at 1600 °C.

The photoluminescence and excitation spectra were measured using a Horiba/Jobin-Yvon Fluorolog-3 spectrofluorometer with a 450 W continuous spectrum xenon lamp for excitation and optical detection with a Hamamatsu R928P photomultiplier operating in the photon counting mode. The measured photoluminescence excitation (PLE) spectra were corrected with the xenon lamp emission spectrum. The luminescence spectra were corrected for the spectral response of the spectrometer system used. The luminescence spectra in the region of R-lines were measured with 0.3-nm spectral resolution and a 0.05-nm increment. The luminescence decay kinetics were measured using the same Fluorolog-3 spectrofluorometer with the excitation light modulated either by a mechanical (chopper) or an electro-magnetic (shutter) modulator. The spectroscopic measurements in the temperature range between about 4 and 325 K were carried out in a Janis continuous-flow liquid-helium cryostat using a Lake Shore 331 temperature controller.

## 4. Results and Discussion

**Figure 1:**
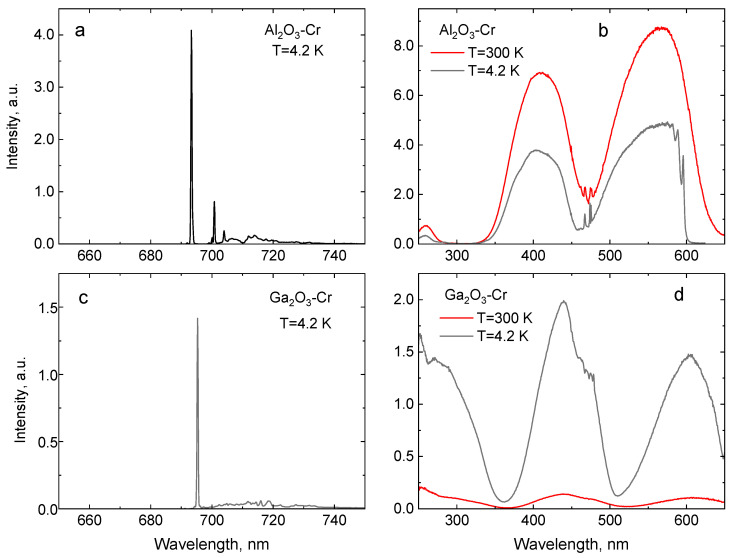
Luminescence (**a**,**c**) and luminescence excitation spectra (**b**,**d**) of Al_2_O_3_-Cr and Ga_2_O_3_-Cr crystals.

**Figure 2:**
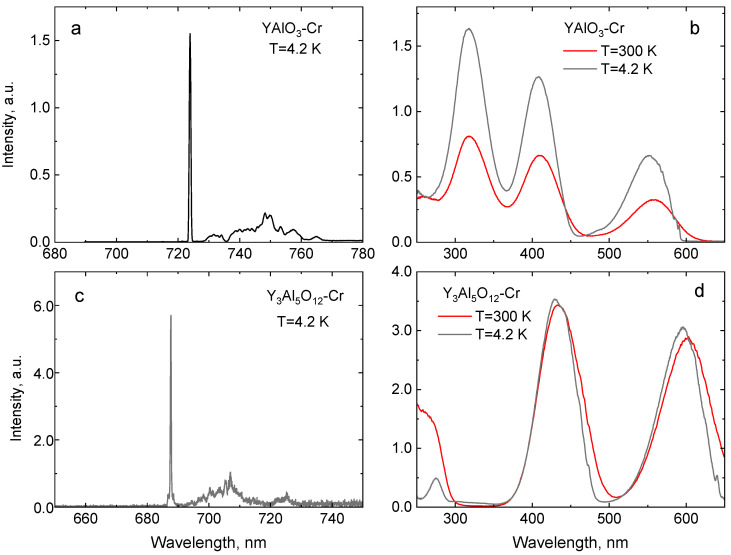
Luminescence (**a**,**c**) and luminescence excitation spectra (**b**,**d**) of YAlO_3_-Cr and Y_3_Al_5_O_12_-Cr.

The use of Cr^3+^ luminescence of doped oxides for temperature monitoring relies on the possibility to achieve good responsivity of the measured emission characteristic to thermal changes. The temperature influences the position and relative occupation probabilities of the levels. These, in turn, are governed by the energy separation of the levels involved. The impact of the crystal field on the energy structure of d^3^-type ions is characterized by the separation of the ^2^E and ^4^T_2g_ levels. This is typically measured by taking into account the difference between the positions of the zero-phonon lines of the ^4^T_2g_ electronic level and the ^2^E level. Thus, it is large for YAlO_3_ and Al_2_O_3_ (around 3100 cm^−1^ and 2350 cm^−1^, respectively), intermediate for Y_3_Al_5_O_12_ (around 1100 cm^−1^), and relatively small, while still positive for Ga_2_O_3_ (around 650 cm^−1^), as can be seen from our measurements presented in Figure 1 and Figure 2. Consequently, dissimilar temperature dependencies are expected in these crystals. In this section, we present the results of our measurements of luminescence and decay time characteristics of Al_2_O_3_-Cr, Ga_2_O_3_-Cr, YAlO_3_-Cr, and Y_3_Al_5_O_12_-Cr as a function of temperature over the 4–300 K range. It should be noted that the extent to which these characteristics are investigated and discussed is determined by the potential for application in non-contact luminescence cryothermometry. A more comprehensive discussion of the optical properties in question can be found elsewhere (see References [39,41,63,64] and also the papers quoted throughout this work).

The luminescence excitation and emission spectra of the crystals under study are shown in Figure 1a,b. The peak positions of the main excitation and emission transitions of Cr^3+^ are summarised in Table 1. 

**Figure 3:**
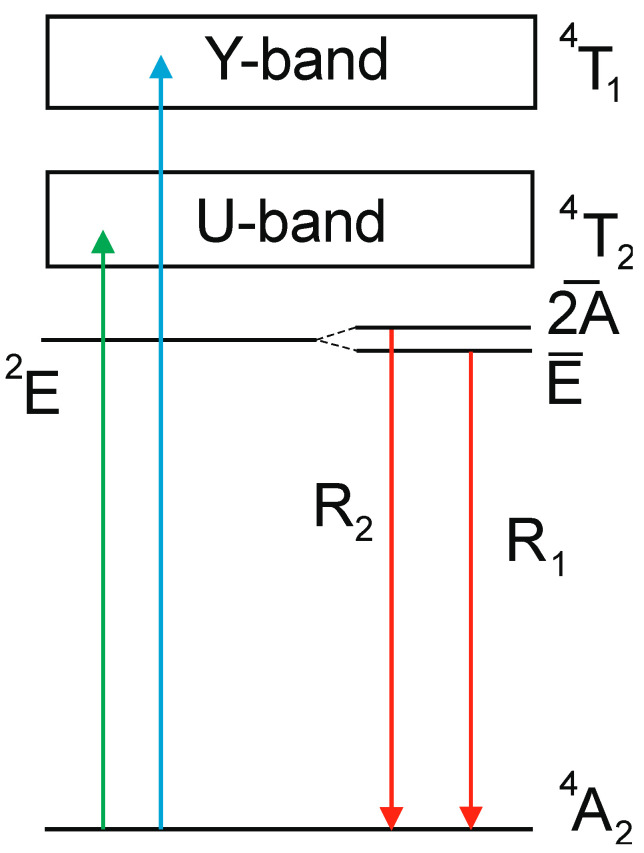
Simplified diagram of energy levels of Cr^3+^ ion in oxide crystals. Vertical arrows show excitation and emission transitions.

A simplified energy diagram of Cr^3+^ transitions [39], displayed in Figure 3, explains the main features of the emission. The luminescence of Cr^3+^ consists of very intense peaks superimposed with a broadband emission in the 650–750 nm spectral range. The two sharp peaks due to spin-forbidden transitions $\bar{E}$→^4^A_2_ and $\bar{2A}$→^4^A_2_ of Cr^3+^ are referred to as the R_1_-lines and R_2_-lines. The broad band is assigned to the phonon-assisted ^4^T_2_→^4^A_2_ transitions. The luminescence excitation spectra consist of two broad excitation bands assigned to the ^4^A_2_→^4^T_1g_ (Y-band) and ^4^A_2_→^4^T_2g_ (U-band) transitions of Cr^3+^ in the 350–500 nm and 500–650 nm wavelength range, respectively. The excitation spectra measured at low temperature exhibit a sharp line structure at the low-energy side of the main bands attributed to the zero-phonon transitions to the ^4^T_1g_, ^4^T_2g_ levels of Cr^3+^, and/or appropriate doublet ^2^T_1g,2g_ levels [63]. The position of these bands is defined by the strength of the crystal field and Racah parameters B and C. The excitation band emerging below 350 nm in Ga_2_O_3_-Cr is due to charge transfer transitions from O^2−^ to Cr^3+^ [65]. Once the photon energy exceeds the band gap energy of the crystal, these are superimposed with the band-to-band transitions, which results in the steep increase in excitation spectra at 260 nm. It should be highlighted that the values of the energy gap between the $\bar{E}$ and $\bar{2A}$ levels on one side and between the ^2^E and ^4^T_2g_ levels on the other define the temperature range in which the luminescence properties of the crystals exhibit a significant change with temperature and, hence, can be used for temperature monitoring. The spectroscopic data indicate that Ga_2_O_3_-Cr is a special case since both values are very different from those observed in other compounds and, hence, one should expect a distinctively different thermometric characteristic at a low temperature.

### 4.1. Temperature Dependence of the Intensity Ratio of Cr^3+^R-lines 

**Figure 4:**
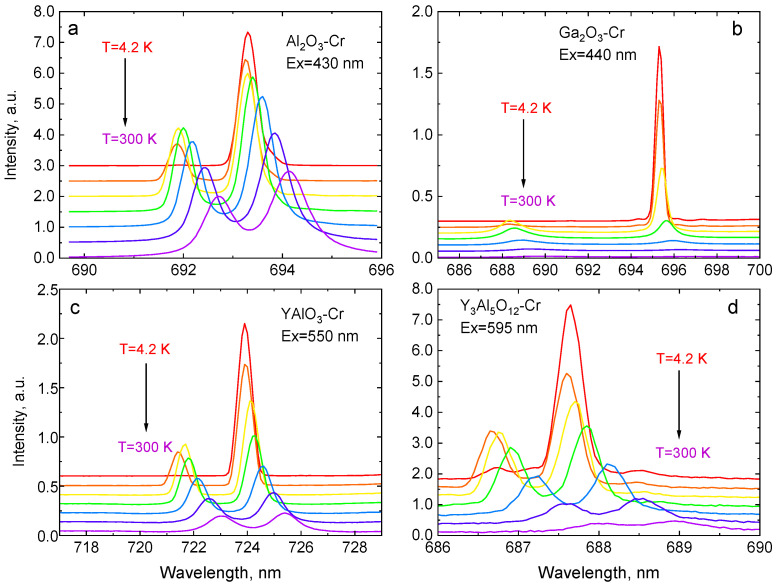
Variation of the luminescence spectra in the region of R-lines with temperature, measured in Cr-doped Al_2_O_3_ (**a**), Ga_2_O_3_ (**b**), YAlO_3_ (**c**), and Y_3_Al_5_O_12_ crystals (**d**). Temperature increment in the plots is 50 K.

**Figure 5:**
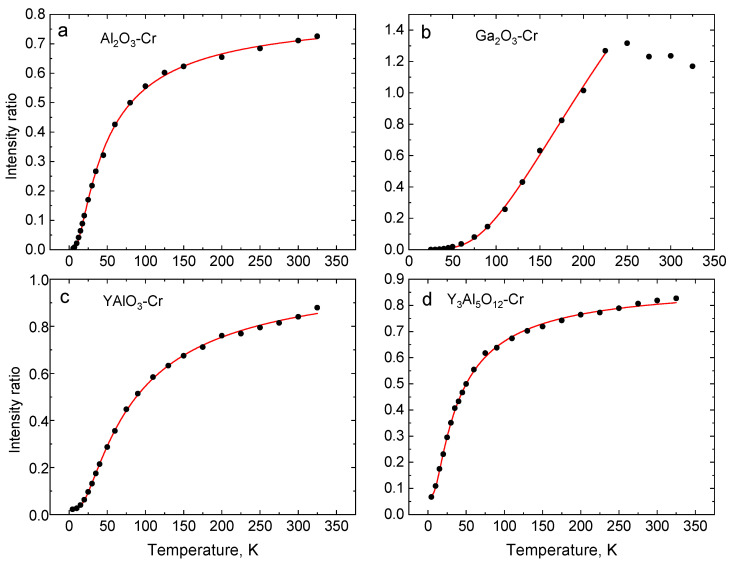
Temperature changes of the luminescence intensity ratio of R-lines ($F=I_{R2}/I_{R1}$) in Al_2_O_3_-Cr (**a**), Ga_2_O_3_-Cr (**b**), YAlO_3_-Cr, (**c**) and Y_3_Al_5_O_12_-Cr (**d**). The red lines show the best fit of experimental results (black dots) to Equation (1) using the parameters presented in Table 2.

The main interest in this study is the change of the emission spectra temperature, specifically changes seen for the R-lines. Figure 3 shows the relevant section of the luminescence spectra of Al_2_O_3_-Cr, Ga_2_O_3_-Cr, YAlO_3_-Cr, and Y_3_Al_5_O_12_-Cr, measured between 4 and 300 K. In all the crystals under study, the Cr^3+^ ion replaces the Al^3+^ or Ga^3+^ in distorted octahedral sites. Due to the distorted octahedral crystal field [39], the lowest excited ^2^E level splits into two levels ($\bar{E}$ and $\bar{2A}$), which gives rise to sharp R-lines in the emission spectra of the crystals. The corresponding states are metastable since the transitions to the ground state are spin forbidden. They are partially allowed due to the admixture of the wavefunction of the ^4^T_2g_ state to that of the ^2^E state and exhibit a long decay time constant (~10^−3^ s). The amount of admixture depends on the magnitude of the spin-orbit interaction and the energy gap between the ^2^E and the ^4^T_2g_ levels, which is proportional to the strength of the crystal field [41,66]. At low temperatures, the metastable levels are populated via a fast relaxation process from the upper-lying ^4^T_2g_ state. The population of the two emitting levels is controlled by processes of electron-phonon interaction between the impurity states and the host lattice. Therefore, in thermal equilibrium, the magnitude of this splitting is the main factor governing the temperature dependence of the luminescence intensity ratio of the two R lines. Due to the small energy gap between the two levels, the effect is particularly noticeable at low temperatures when the intensity of the R_1_-lines drastically increases while it decreases for R_2_, as shown in Figure 4. This effect is visualised in Figure 5 where the temperature variation of the intensity ratio of the two lines in the crystals under study is displayed.

The position of the emission spectra of the crystals reveals two distinct features, which is a significant shift of the R-lines toward the red in YAlO_3_-Cr and a much larger split between R-lines in Ga_2_O_3_-Cr in comparison with Al_2_O_3_-Cr. The variation of position of the R-line in different hosts is readily explained by the nephelauxetic effect [67]. Delocalization of the *d*-orbitals of Cr^3+^ due to the formation of chemical bonds with ligands decreases the energy of the ^2^E states. In perovskite-type YAlO_3_ crystals with orthorhombic structure, the Cr^3+^ substitute Al^3+^ in the slightly distorted octahedra AlO_6_, is compressed along the c-axis and stretched along the b-axis [68]. This causes a strong nephelauxetic effect and a shift of the R-lines in YAlO_3_-Cr toward lower energies [69,70]. Lastly, the emission of Cr^3+^ in Y_3_Al_5_O_12_ exhibits the smallest split and low-energy shift of R-lines. This is indicative of a weaker distortion from ideal octahedral symmetry.

The greater energy separation between the $\bar{E}$ and $\bar{2A}$ levels in Ga_2_O_3_ is explained by lower site symmetry of the Cr^3+^ ion in the host compared with Al_2_O_3_ [70,71,72]. This is consistent with the results of electron paramagnetic resonance (EPR) studies of Ga_2_O_3_-Cr [73], which demonstrates a larger energy splitting of the ground ^4^A_2_ level of Cr^3+^ in Ga_2_O_3_ (1.16 cm^−1^) when compared with that of Al_2_O_3_ (0.38 cm^−1^) due to a lowering of the local symmetry from trigonal to monoclinic. This translates to a visibly dissimilar temperature dependence of the emission properties. On the one hand, because of the larger energy gap, the phonon-induced population of the upper $\bar{2A}$ level in Ga_2_O_3_-Cr ceases at T < 50 K, which is at a much higher temperature in comparison with Al_2_O_3_-Cr. On the other hand, thermal quenching of the Cr^3+^ emission in Ga_2_O_3_ starts at much lower temperatures (ca. 200 K). This causes a more pronounced variation of the intensity ratio of the two lines over a narrower temperature range, which is displayed in Figure 4. Both effects eventually lead to the higher temperature sensitivity of Ga_2_O_3_-Cr over this temperature range.

The population process of the two levels in thermal equilibrium obeys Boltzmann statistics, which gives the following expression for the luminescence intensity ratio.

(1) $$F\left(T\right)=Aexp\left(-\frac{D}{kT}\right)+B$$

In this case, *A* is the fitting parameter dependent on the spontaneous emission rate, its frequency and degeneracy of two levels, D is the energy difference between two emitting levels, k is the Boltzmann constant, T is the absolute temperature, and B is a constant offset. The latter accounts for the background contribution to the signal originating from other emission processes [74]. The important advantage of using the intensity ratio as a measure for temperature of a thermally coupled system is that the population of the individual levels is directly proportional to the total population. The change in total population due to a variation of excitation affects the population of the individual level to the same extent, which renders the luminescence intensity ratio independent of excitation intensity. Formula (1) was used to fit the experimental results and derive the calibration curve (see Figure 5). The values of the fitting parameters are summarised in Table 2. 

It can be seen that, for Cr-doped Al_2_O_3_, YAlO_3_, and Y_3_Al_5_O_12_, the value for the energy gap between the two levels derived from the fitting correlates very well with the results of spectroscopic studies (Table 1). For Ga_2_O_3_-Cr, this is not the case. In this case, the fitted value is ca. 50% higher. This is indicative of energy transfer between two thermalized levels and another state [21]. To understand this discrepancy, one needs to bear in mind the quantitative difference in the energy structure of Cr^3+^ in this host leading to such changes. The energy gap between the $\bar{E}$ and $\bar{2A}$ levels in Ga_2_O_3_ is only by about a factor 4 less than the energy difference between the ^2^E and ^4^T_2_ [71,75] whereas, in other crystals of this study, this ratio is more than 25. Because of this, the $\bar{E}$ and $\bar{2A}$ levels of Cr^3+^ in Ga_2_O_3_ cannot be treated as an isolated system at low temperatures. Basically, at temperatures above ca. 50 K, when the upper $\bar{2A}$ level is sufficiently populated to give measurable luminescence (see Figure 4b) the probability of the depopulation through ^2^E→^4^T_2_ transitions is not negligible again. This additional channel is the reason for the observed overestimated value of the energy gap D in the crystal, as obtained from fitting.

### 4.2. Wavelength Shift of Cr^3+^ R-Lines with Temperature 

**Figure 6:**
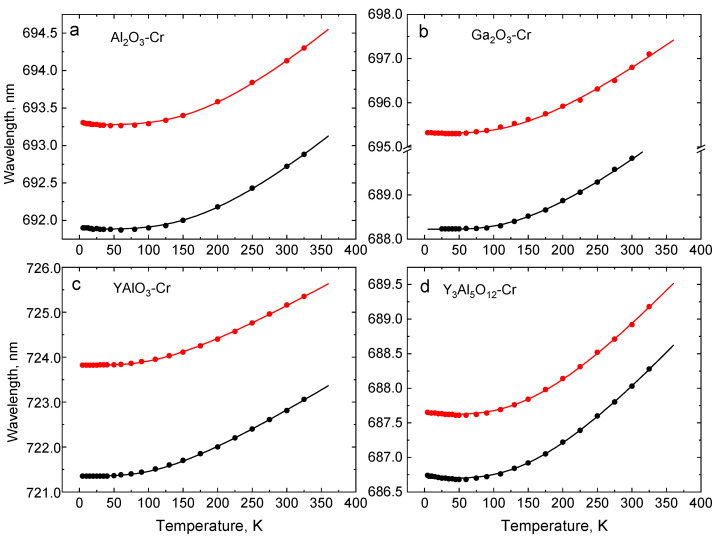
Positions of the R_1_- (red) and R_2_- (black) lines in Al_2_O_3_-Cr (**a**), Ga_2_O_3_-Cr (**b**), YAlO_3_-Cr (**c**), and Y_3_Al_5_O_12_-Cr (**d**) as a function of temperature. The dots are experimental data points and the curves show the best fit of experimental results for Equation (2), using parameters presented in Table 2.

The absolute wavelength shift of the R-lines Δν is yet another feature of the Cr^3+^ emission that can be readily used in thermometry. Figure 6 displays the shift induced by temperature in the crystals under study. The mechanism of the effect is explained in terms of the interaction of electron states of the impurity ion with the phonons of the host lattice via absorption/emission of a single resonant phonon and a Raman process involving scattering of two phonons [39]. The theoretical interpretation of the wavelength shift with temperature has been developed by McCumber and Sturge and applied to Al_2_O_3_-Cr emission [42]. According to the model, the thermal shift of the lines Δν can be approximated by the following equation.

(2) $$\Delta \nu \left(T\right)=\alpha {\left(\frac{T}{T_{D}}\right)}^{4}\overset{T_{D}/T}{\underset{0}{\int }}\frac{x^{3}}{\exp\left(x\right)-1}dx$$

where α is the coupling coefficient for the electron-phonon interactions and $T_{D}$ is the Debye temperature of the material. The formula was used as the model for a correlated fit of the thermal shifts of the R-lines, which allows individual α for the two lines, individual values for wave numbers at the lowest temperatures, and the Debye temperature $T_{D}$ common to both (R_1_ and R_2_) data sets. Very good fit results were achieved for the crystals studied, in this case, throughout the entire measurement range. The fit parameters are summarised in Table 3. Note that an earlier study of the wavelength shift in Al_2_O_3_-Cr yielded $T_{D}=$ 750 K [42], while the Debye temperature derived from the fit in this scenario is closer to the consolidated reference value ($T_{D}=$970 K [76]). The values of Debye temperatures obtained from the fit for YAlO_3_ and Y_3_Al_5_O_12_ are in good agreement with that derived from measurements of temperature changes of crystal structures ($T_{D}=$ 534 K [77] and 760 K [78], respectively). Lastly, the fitted value of the Debye temperature for Ga_2_O_3_ ($T_{D}=$ 682 K) is consistent with that obtained from a measurement of the thermal conductivity ($T_{D}=$ 738 K) [79]. 

Inspection of the data presented in Figure 6 shows that the wavelength shift of the R-lines is insignificant at low temperatures. In YAlO_3_-Cr and Ga_2_O_3_-Cr, the lines start to exhibit a recognizable departure from a near constant at T > 70 K, while, in Cr-doped Al_2_O_3_ and Y_3_Al_5_O_12_, the onset of the changes is at an even higher temperature (ca. 100 K). This defines the lower limit of a temperature range where each crystal can be used for temperature monitoring based on the wavelength shift of R-lines.

### 4.3. Temperature Dependence of the Luminescence Decay Time 

**Figure 7:**
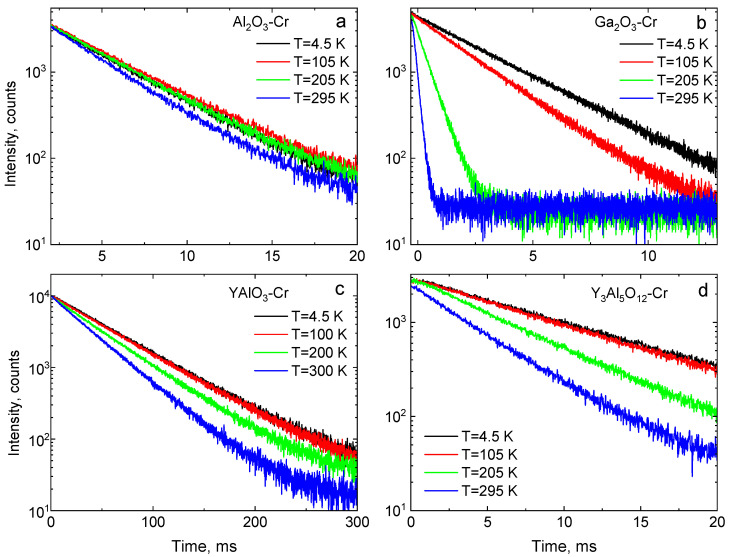
The luminescence decay curves of Cr^3+^ emissions in Al_2_O_3_-Cr (**a**), Ga_2_O_3_-Cr (**b**), YAlO_3_-Cr (**c**), and Y_3_Al_5_O_12_-Cr (**d**).

**Figure 8:**
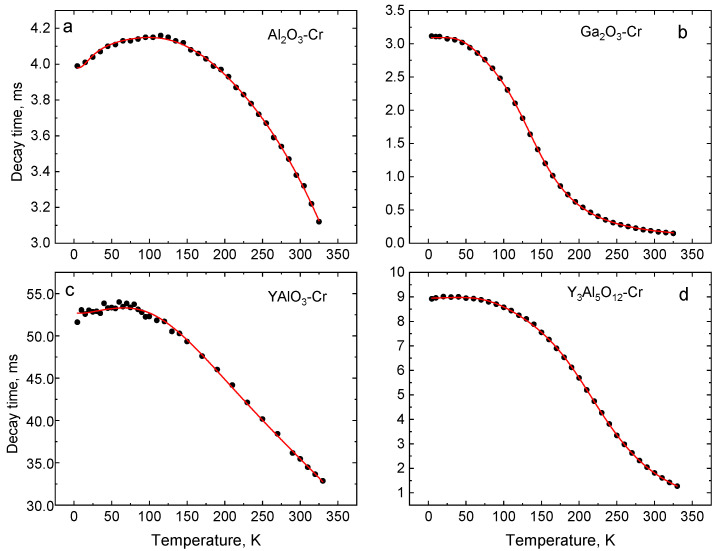
Temperature dependence of the luminescence decay time constant of Cr^3+^ emission in Al_2_O_3_-Cr (**a**), Ga_2_O_3_-Cr (**b**), YAlO_3_-Cr (**c**), and Y_3_Al_5_O_12_-Cr (**d**). The curves show the best fit of Equation (5) to the experimental results with the fit parameters presented in Table 4.

The variation of luminescence decay time constants of the R-lines of Cr^3+^ over the 4–325 K temperature range was measured to evaluate the decay-time-based responsivity characteristics of the crystals. Decay curves at different temperatures are shown in Figure 6. The emission process in Cr^3+^ can be described by a classic model of radiative decay of isolated impurity centres. The resultant exponential decay curve for the intensity I(t)  is $I_{0}\exp\left(-t/\tau \right)+C$, where$\;I_{0}$ is the initial intensity, τ is the luminescence decay constant, and C is the background [39] (see Figure 7). The decay time constants as a function of temperature determined from the data in Figure 7 are plotted in Figure 8. The common trend observed in all crystals is an increase of the decay time constants with cooling. However, the gradients in τ=f(T) are unique to each material. The main differences in the τ=f(T) plots are observed at low temperatures (see Figure 7). For example, in Al_2_O_3_-Cr, at temperatures below ≈100 K, the decay time constants shorten when cooling, rather than continuing the trend seen at higher temperatures. A similar, though less pronounced, decrease in the temperature dependence of the decay time constant of Y_3_Al_5_O_12_-Cr is seen below 20 K. In YAlO_3_-Cr, we also detected a small yet steady decrease of the decay time constant below 70 K. Ga_2_O_3_-Cr is the sole material where τ=f(T) keeps increasing with cooling until it reaches a steady value.

Such a significant difference in the behaviour of the Cr^3+^ emission τ=f(T) characteristic, especially when individual to the material and prominent at low temperatures, is intriguing and deserves further investigation. The earlier explanation of this effect [40,80] suggests that the decrease in τ=f(T) of Al_2_O_3_-Cr below 100 K (see Figure 8a) is due to thermally-activated transitions from the lower $\bar{E}$ to the upper $\bar{2A}$ level. Since the upper level has a longer radiative lifetime, such transitions cause the increase of the measured decay constant. At further cooling, the probability of such a transition and, hence, population of the upper level is gradually reduced, which translates into a decrease of the decay time constant. This is then determined predominantly by the radiative rate of the lower level. It is readily understood, therefore, that the magnitude of the energy split of the emission states of Cr^3+^ and the respective transition rates are the main factors controlling the shape of the τ=f(T) characteristic at low temperatures.

This interpretation envisions that a thermally activated exchange between the split levels should manifest itself in a temperature dependence of the luminescence decay time in all Cr-doped compounds. So far, this has been reported and discussed only for ruby [40], and alexandrite [81]. It seems plausible that, in other materials, other effects may mask it. One such example is Y_3_Al_5_O_12_-Cr. The decrease of the decay time constant at low temperature observed by us is prominently visible (see Figure 8d), whereas earlier studies missed this feature [82,83], which leads to an alternative interpretation of the temperature dependency. Another example is YAlO_3_-Cr, which exhibits the longest decay time constant (55 ms) in comparison with other Cr-doped compounds under study. Due to this, the relative decrease of the decay time constant at low temperatures accounts for ca. 5% (see Figure 8c), which is easy to overlook or even attribute to experimental errors. In Ga_2_O_3_-Cr, the luminescence decay time constant exhibits a steady rise with a decreasing temperature (see Figure 8b). One possible explanation of this behaviour is that, due to a large energy splitting of the ^2^E level and the low thermal quenching temperature, the thermal activation of the upper $\bar{2A}$ level is superseded by the thermal depopulation through the ^2^E→^4^T_2_ transitions. Another possibility to explain this observation is to assume the opposite relationship between the radiative rates of two levels in the crystal, i.e., the radiative lifetime of the lower level is longer than that of the upper level. For temperature monitoring, a monotonic change of the decay time constant is preferable. Thus, it would be very helpful to get better insight into the mechanism behind this behaviour. Motivated by this, we developed a phenomenological model that can provide an interpretation for the variety of τ=f(T) characteristics, which is measured here.

The basis and prerequisite of the model is that the impurity ions of Cr^3+^ strongly interact with the crystal field and lattice vibrations of the host. Consequently, changes in temperature that alter the crystal field, phonon density, and their energy affect the emission decay time constant. The temperature dependence of the luminescence decay time constant of Cr^3+^ can be described by a model that takes into account two types of processes affecting the population of the ^2^E levels, i.e., thermally-induced depopulation and phonon-assisted relaxation of the emission centre. With the assumption that the states are in thermal equilibrium, the observed decay time will be a thermal average of the radiative decay rates of the three levels ($\bar{E}$, $\bar{2A},$ and ^4^T_2_) involved in the transitions, and weighted by the Boltzmann occupation factor and degeneracy.

(3) $$\frac{1}{\tau \left(T\right)}=\frac{\sum_{i=1}^{3}g_{i}R_{i}exp\left(-\frac{\Delta E_{i}}{kT}\right)}{\sum_{i=1}^{3}g_{i}exp\left(-\frac{\Delta E_{i}}{kT}\right)}$$

In this expression, $R_{i}$ are the radiative decay rates, $g_{i}$ are the degeneracies of the states, and $\Delta E_{i}$ is the energy difference between the ith state and the lower excited level. In this case, the integers i = 1, 2, and 3 correspond to the $\bar{E}$, $\bar{2A}$, and ^4^T_2_ levels having a degeneracy equal to 2, 2, and 12, respectively. Subsequently, $\Delta E_{1}$= 0, $\Delta E_{2}\;$= D, the energy split of the ^2^E levels, and $\Delta E_{2}$ = ΔE is the energy difference between the ^2^E and ^4^T_2_ levels. 

Another effect causing a change of the radiative decay rates with temperature is phonon-induced interactions. This effect can be accounted for by a factor $coth\left(E_{p}/2kT\right)$, [39,82,84], resulting in the following expression for the radiative decay rate of the ith state.

(4) $$R_{i}=\frac{1}{\tau_{i}}coth\left(\frac{E_{p}}{2kT}\right)$$

where $\tau_{i}$ is the radiative decay time constant, and $E_{p}$ stands for “effective energy” of the phonons responsible for the exchange with the sidebands. In the following derivation, we introduce two simplifications. First, because of the proximity of the $\bar{E}$ and $\bar{2A}$ levels, we assume that the same value for the phonon energy $E_{p}$ can be used to characterise the phonon-induced interaction with both levels. Second, we omit the phonon-induced interaction for the radiative decay transitions occurring from the ^4^T_2_ level and use a temperature-independent term $R_{3}=1/\tau_{3}$. This is a valid approximation since these transitions become involved only in the radiative decay process of Cr^3+^ at higher temperatures when thermally assisted depopulation dominates the phonon-mediated exchange with sidebands. Thus, the expression for τ(t) can be rearranged as follows.

(5) $$\tau \left(T\right)=\frac{1+exp\left(-\frac{D}{kT}\right)+6exp\left(-\frac{\Delta E}{kT}\right)}{\frac{1}{\tau_{1}}coth\left(\frac{E_{p}}{2kT}\right)+\frac{1}{\tau_{2}}coth\left(\frac{E_{p}}{2kT}\right)exp\left(-\frac{D}{2kT}\right)+\frac{6}{\tau_{3}}exp\left(-\frac{\Delta E}{kT}\right)}$$

In this case, $\frac{1}{\tau_{i}}=R_{i}$ (i = 1, 2 and 3) are the radiative decay rates of the $\bar{E}$, $\bar{2A}$, and ^4^T_2_ levels, respectively. This equation accounts for the thermalisation process occurring between the $\bar{E}$ and $\bar{2A}$ levels and phonon-assisted relaxation and depopulation of the levels due to thermally induced ^2^E→^4^T_2_ transitions. These are the main processes that determine the τ=f(T) behaviour in the low-temperature region. 

This model (Equation (5)) was then used to fit the experimental data for Al_2_O_3_-Cr by achieving good agreement of experimental data and theory over the entire temperature range (see a solid line in Figure 7a). The parameters of the fit are summarised in Table 4. A very similar model had been adopted by Zhang et al. [40] to fit the temperature dependence of the decay time of Al_2_O_3_-Cr. The main difference is the number of free parameters used for the fit. In Reference [40], two fixed values for the phonon energies $E_{p}$ and the ratio $\tau_{1}/\tau_{2}$ were used while, in the case presented here, all parameters except the energy gap between the two levels, D, are free parameters, which result in greater accuracy of the parameters. 

The relation between the radiative lifetimes of the two levels (<1), as obtained, is consistent with the reasoning proposed in this case for the interpretation of the observed change of the luminescence decay time constant at low temperatures. This relation between $\tau_{1}\;\mathrm{and}\;\tau_{2}$, the population of the upper $\bar{2A}$ level occurring at the expense of depleting the lower emission level of Cr^3+^, leads to the increase of the measured decay time of Al_2_O_3_-Cr with an increase of temperature over the 4–100 K range.

We then applied the model to fit the measured temperature dependencies of the decay time of other crystals and obtained exceptionally good agreement with the experimental results (see Figure 8b–d). The parameters of the fits, i.e., effective phonon energies and activation energies of thermal quenching, correlate with those quoted for the emission of the crystals in References [72,82,85]. Similar to the ruby, the radiative lifetime of the upper level $\tau_{2}$ of Cr^3+^ in YAlO_3_ and Y_3_Al_5_O_12_-Cr is higher than for the lower level, which explains the decrease of the decay time constant with cooling below 70 K and 20 K, respectively. 

The fitting of the τ=f(T) dependency of Ga_2_O_3_-Cr yielded the most interesting result. The lifetime of the lower level exceeds the respective value for the upper level by a factor of three, which results in an inverse relation of the two parameters ($\tau_{1}/\tau_{2}$ > 1). This has major implications for the temperature dependence of the decay time constant. Since the lower level $\bar{E}$ has a longer radiative lifetime, the thermal activation of the upper $\bar{2A}$ level at heating results in a steady increase of the overall transition rate that, in turn, translates into a monotonic decrease of the decay time constant with temperature in Cr-doped Ga_2_O_3_. 

For thermometric applications, this type of temperature dependency is much preferred. This is in contrast to the situation observed in other crystals where the slope of τ=f(T) plot changes its sign at around 120 K (Al_2_O_3_-Cr), 70 K (YAlO_3_-Cr), and 20 K (Y_3_Al_5_O_12_-Cr), which causes ambiguity of the temperature reading and much reduced sensitivity in the important region of cryogenic temperatures. In the next section, we will discuss the effect different features of luminescence of Cr-doped oxides have on the performance characteristics of temperature sensing methods. 

## 5. Comparison of Temperature Sensing Schemes 

Having investigated the temperature changes of different luminescence parameters in Cr-doped oxides and determined the analytical expression for the dependence, we can evaluate and compare the merit of these materials for non-contact thermometry when used in different modes of temperature sensing. The sensitivity S of different materials can be defined as the modulus of the fraction of change of measured thermometric parameter Q-either luminescence intensity ratio, wavelength shift, or decay time constant- over changes with temperature.

(6) $$S=\left|\frac{dQ}{dT}\right|$$

For good sensitivity over a wide range of temperatures, a large value of the derivative |dQ/dT| over the full range is desirable. This parameter alone cannot, however, be used for direct comparison of different methods for temperature sensing as it is expressed in different units, depending on the monitored variable. One way to regulate the comparison throughout different materials and temperature measurement methods is to use the temperature uncertainty or resolution δT, which can be calculated as a ratio of experimental error of the measured parameter δQ and sensitivity [8].

(7) $$\Delta T=\Delta Q{\left|\frac{dQ}{dT}\right|}^{-1}$$

This parameter represents the smallest temperature difference that can be resolved by the sensor and, hence, can serve as an indicator of the measurement accuracy [86]. 

To determine this parameter, one needs to have a reasonable estimate for the standard error on the mean of the measured intensity ratio, decay time, and wavelength shift. The relative errors for measurements of decay time and intensity ratio over the examined temperature range are estimated to be 0.5% and 2%, respectively, while the absolute error in determining the wavelength shift is taken as a temperature-independent constant value equal to 0.02 nm. Expression (7) and the temperature dependencies of the measured parameters were then used to calculate the uncertainty of temperature measurements as a function of temperature. 

**Figure 9:**
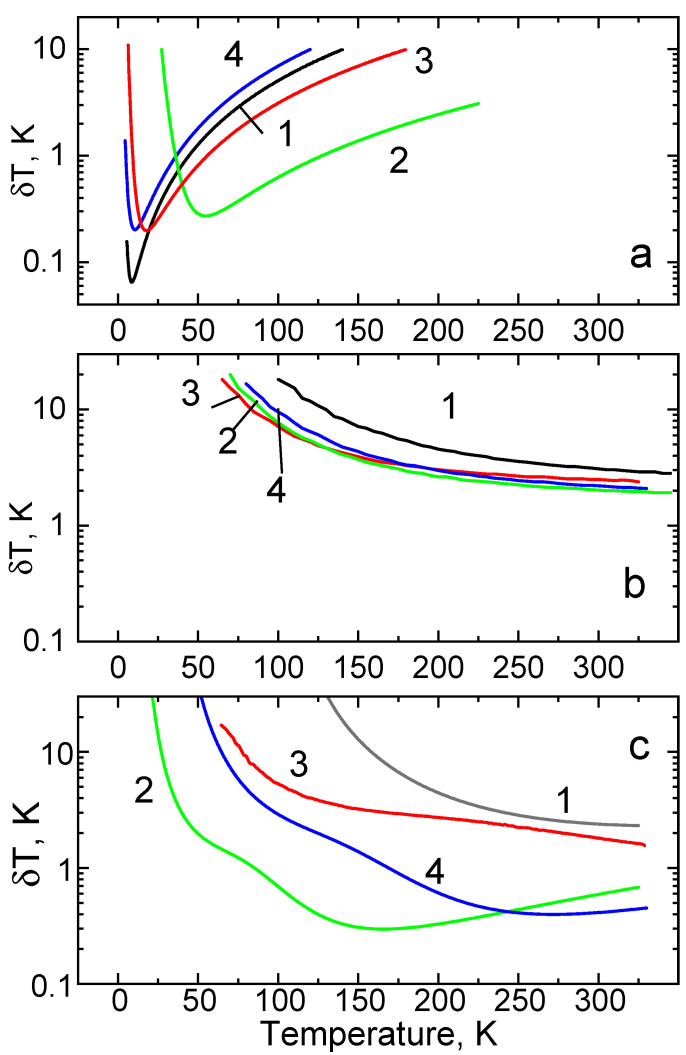
The temperature resolution of Al_2_O_3_-Cr (1, black), Ga_2_O_3_-Cr (2, green), YAlO_3_-Cr (3, red), and Y_3_Al_5_O_12_ (4, blue) for different modes of temperature sensing: (**a**)—luminescence intensity ratio, (**b**)—wavelength shift, (**c**)—decay time constant, where the curves are calculated using the fitted τ=f(T) dependence and measurement uncertainties.

The temperature dependence of the thermometric resolutions for the crystals discussed here are presented in Figure 9a–c. All the plots demonstrate a significant (in some cases, more than two orders of magnitude) variation of δT over the temperature range of interest. To compare the relative sensitivity of different crystals and in different regimes of temperature measurements, we use a value of δT = 1 K as a separator between high and low resolution of a temperature sensor. The temperature resolution typically reported for non-contact luminescence sensors using decay time and intensity ratio techniques can vary over a broad range from 5 × 10^−3^ to 5 K [2,6,8,9,23,27,38,56,74,87,88,89,90].

Inspection of the δT=f(T) plots shows that good temperature resolution over the 5–100 K range can be achieved by monitoring the luminescence intensity ratio of the R-lines in Cr^3+^-doped oxides (see Figure 9a). The best value of temperature uncertainty, ±0.06 K at 10 K, observed for Al_2_O_3_-Cr is superior to what is typically found in other materials [2,8]. The underlying mechanism, which leads to good temperature sensitivity of the intensity ratio method, is a low energy gap between the two levels involved in the luminescence process. However, the resolution of Al_2_O_3_-Cr diminishes very significantly with an increasing temperature so that, at above 25 K, YAlO_3_-Cr is better. Ga_2_O_3_-Cr, which possesses the largest energy gap between the $\bar{E}$ and $\bar{2A}$ levels of Cr^3+^, manifests the best temperature resolution (better than ±1 K) over the 40–140 K range. The use of the intensity ratio of R-lines in Ga_2_O_3_-Cr for temperature reading has, however, one important shortcoming, limited operation range of sensing. On the one hand, because of the large energy difference between the two states, the population of the upper levels swiftly drops with cooling, and, thereby, limits the ability to determine temperature. On the other hand, due to strong thermal quenching, the intensity of the R-lines decreases rapidly with increasing temperature, which leads to a concomitant decrease of the signal-to-noise ratio, which renders a temperature evaluation above 200 K difficult.

Temperature monitoring using the wavelength shift of the R-lines of Cr^3+^ luminescence exhibits the worst temperature resolution, as shown in Figure 9b. This is due to the low sensitivity of the method. For example, at T > 200 K, the peak wavelength of the R_1_ line of Cr^3+^ shifts with a gradient of ca. 1 × 10^−2^ nm/K [43] whereas the accuracy of determining the position of the sharp R-lines is ±0.02 nm. Furthermore, the slope of Δν=f(T) rapidly reduces with cooling, which translates to poor accuracy of the temperature reading at lower temperatures. Therefore, this method is competitive merely at higher temperatures where the uncertainty reduces to a few K. At 300 K, the temperature resolution is ±3 K for Al_2_O_3_-Cr and ±2 K for Ga_2_O_3_-Cr while YAlO_3_-Cr and Y_3_Al_5_O_12_-Cr are in between the two. 

The last plot (Figure 9c), which shows the temperature resolution of the decay time thermometry achievable with Cr^3+^ doped crystals, reveals the most interesting features. Al_2_O_3_-Cr, which has been extensively promoted for high-temperature applications, exhibits an increase of the uncertainty at cryogenic temperatures. The best temperature resolution at 300 K, δT= ±2.5 K, is consistent with previously reported values [50], but it deteriorates significantly with cooling. Therefore, in case of Al_2_O_3_-Cr, decay time thermometry is inferior when compared to the intensity ratio technique for applications below room temperature. Despite better performance of YAlO_3_-Cr, the accuracy of measurements at low temperatures is also limited. The longer decay period is another disadvantage of this material, which makes measurements based on this approach longer. This is not ideal for practical applications. Inspection of the data in Figure 9c shows that Y_3_Al_5_O_12_-Cr exhibits significantly better temperature resolution than the former two compounds, but, evidently, it is Ga_2_O_3_-Cr that represents the best material for decay time thermometry at cryogenic temperatures. The crystal exhibits good temperature resolution (better than ±1 K) over a broad range (80–300 K). The lowest uncertainty of the temperature measurements is found to be ±0.3 K at 165 K, which is comparable to the accuracy of standard resistance temperature sensors and the best resolution achieved by non-contact luminescence decay time sensors [9,23]. It appears that Cr-doped Ga_2_O_3_, applied to temperature sensing, is superior in comparison with the other two crystals. Overall, these findings correlate very well with the earlier reasoning regarding the impact of the energy structure of Cr^3+^ on the thermometric sensitivity of the decay time method in the temperature range in question. The relatively small energy gap between the ^2^E and ^4^T_2g_ levels in materials with intermediate crystal field strength is beneficial for cryogenic thermometry while hosts with a larger energy gap are more suited for measurements at higher temperatures.

## 6. Conclusions 

The sensitivity of Cr^3+^ luminescence to changes of temperature is put to use for non-contact measurements of temperature by monitoring the relevant emission characteristics, namely, intensity ratio of R-lines, wavelength shift, and decay time constant. Renewed interest in non-contact luminescence sensing is prompted by the necessity to monitor the temperature of cryogenically-preserved proteins and cells subjected to exposure of ionising radiation in X-ray crystallography, X-ray, and electron microscopy or tomography experiments. We evaluated the feasibility and potential of Cr-doped Al_2_O_3_, Ga_2_O_3_, YAlO_3_, and Y_3_Al_5_O_12_ for temperature sensing over the 4–300 K range. 

The main step forward presented in this case with respect to what has previously been reported for some of the materials in the context of non-contact luminescence sensing of temperature is an extended examination of luminescence characteristics and identification of principal features influencing thermometry over the above temperature range. This is complemented by comprehensive analysis and interpretation of thermometric properties of the crystals under study. To do this, we applied pertinent theoretical models used to explain the observed temperature dependencies by changes in the dynamics of the excited level populations due to (i) thermalization, (ii) electron-phonon interaction, (iii) non-radiative multi-phonon relaxation, and (iv) thermal depopulation of higher states. The intensity ratio of R-lines at low temperatures is predominantly controlled by the process (i) responsible for redistribution of populations between the upper and lower levels. The process (ii) is the cause of a wavelength shift while the explanation of the temperature change of the decay rate needs to take into account the combined effects of all processes (i)−(iv). We then demonstrated that the models provide adequate interpretation of the processes, which can be judged from the high quality of fits obtained for the measured temperature dependence of the luminescence intensity ratio F, wavelength shift Δν, and decay time constant τ in the crystals under study. The results of the fits yielded a number of quantitative parameters of which further analysis allows us to establish how the energy structure of the emission centre and phonon dynamics in different hosts influence the luminescence properties and what effect it has upon the different modes of non-contact luminescence temperature sensing. We demonstrated that the temperature dependence of the luminescence decay time in Al_2_O_3_-Cr, YAlO_3_-Cr, and Y_3_Al_5_O_12_-Cr exhibits qualitatively different behaviour to that of Ga_2_O_3_-Cr. According to the model that describes the dynamics of the luminescence process of Cr^3+^, this is due to the change in the ratio of radiative decay rates of $\bar{E}$ and $\bar{2A}$ levels ($\tau_{1}/\tau_{2}$). The ratio is found to be less than one in all crystals but Ga_2_O_3_-Cr, where $\tau_{1}/\tau_{2}$ > 1.

Lastly, we evaluated the thermometric performance of the three methods of temperature sensing in four crystals. The comparison shows that the intensity ratio method has high accuracy and, hence, it can be used at very low temperatures. It is found that Ga_2_O_3_-Cr, which possesses the largest energy gap between the emitting levels of Cr^3+^, manifests good temperature resolution (±1 K or better) over the 40–140 K range. This is contrasted by Al_2_O_3_-Cr and YAlO_3_-Cr where a similar level of temperature resolution is observed below 50 K. The spectral line shift technique has not proven to be adequate for temperature sensing in the range of cryogenic temperatures since all the materials exhibit relatively small shifts of the Cr^3+^ emission lines and, subsequently, exhibit large measurement uncertainties. The study also demonstrated that the non-contact decay time thermometry using Al_2_O_3_-Cr and YAlO_3_-Cr is suboptimal for application at low temperatures. The temperature resolution is ca. ±2.5 K at 300 K and it degrades further with cooling. In contrast, Y_3_Al_5_O_12_-Cr is well suited for temperature monitoring when using this method at above 150 K. Best performance can be achieved with Ga_2_O_3_-Cr. The crystal exhibits resolution better than ±1 K over a broad temperature range (80–300 K). This demonstrates that, among the examined materials, Ga_2_O_3_-Cr shows considerable promise for the technology of non-contact thermometry sensing. The full complement of Ga_2_O_3_-Cr capabilities as a non-contact cryogenic temperature sensor includes performance comparable or superior to other sensors and a possibility to use two sensing modes that offer comparable resolution in the range of cryogenic temperatures. The results of this study unequivocally demonstrated that Ga_2_O_3_-Cr is the preferred material for non-contact temperature monitoring within the cryogenic range 30–200 K that is of primary interest for experimenting with frozen proteins and cells. 

## Figures and Tables

**Table 1:** Position of the main Cr^3+^ emission peaks and excitation bands in the measured crystals at 4 K.

Crystal	Band Gap, eV	$\bar{E}\to^{4}A_{2}(R_{1}-\mathrm{Line}),\;\mathrm{nm}$	$\bar{2A}\to^{4}A_{2}(R_{2}-\mathrm{Line}),\;\mathrm{nm}$	R_1_-R_2_ Gap, meV	^4^A_2_→^4^T_1g_ (Y-BAND), nm	^4^A_2_→^4^T_2g_ (U-Band), nm	^2^E-^4^T_2g_ Gap, meV
Al_2_O_3_	9.4	693.3	692.7	3.6	411	568	290
Ga_2_O_3_	4.85	695.3	689.7	18.2	441	608	80
YAlO_3_	8.5	723.9	723.0	5.9	422	564	380
Y_3_Al_5_O_12_	7.1	687.7	688.9	2.4	434	601	135

**Table 2:** Parameters of fits obtained from the temperature dependence of the luminescence intensity ratio, Equation (1), for Cr-doped oxides investigated in this work.

Parameter	Al_2_O_3_-Cr	Ga_2_O_3_-Cr	YAlO_3_-Cr	Y_3_Al_5_O_12_-Cr
A	0.809 ± 0.004	5.39 ± 0.19	1.019 ± 0.004	0.814 ± 0.008
D, meV	3.36 ± 0.04	28.4 ± 0.6	5.8 ± 0.1	2.8 ± 0.1

**Table 3:** Parameters of fits obtained from the temperature dependence of the luminescence wavelength shift in Equation (3) for Cr-doped oxides investigated in this work.

Parameter	Al_2_O_3_-Cr	Ga_2_O_3_-Cr	YAlO_3_-Cr	Y_3_Al_5_O_12_-Cr
$\alpha_{R1}$, cm^−1^	−518.0	−534.2	−284.5	−570.7
$\alpha_{R2}$, cm^−1^	−507.5	−582.8	−317.8	−581.1
T_D_, K	867	682	541	739

**Table 4:** Parameters of fits obtained from the temperature dependence of the luminescence decay time constant (Equation (5)) for Cr-doped oxides.

Parameter	Al_2_O_3_-Cr	Ga_2_O_3_-Cr	YAlO_3_-Cr	Y_3_Al_5_O_12_-Cr
τ_1_, ms	3.98 ± 0.01	3.09 ± 0.01	52.6 ± 0.2	8.94 ± 0.03
τ_2_, ms	4.46 ± 0.01	1.02 ± 0.04	55.4 ± 1.0	9.04 ± 0.08
E_p_, meV	60.9 ± 0.4	35.7 ± 0.3	41.4 ± 0.6	32.6 ± 0.3
*D* *, meV	3.6	18.2	5.9	2.4
τ_3_, μs	33 ± 25	30.1 ± 1.4	263 ± 28	32.6 ± 0.3
Δ*E*, meV	237 ± 23	83 ± 1	279 ± 36	138 ± 0.7

* The value of D is fixed to be equal to the energy splitting of the ^2^E level.
